# Phenotypic and genotypic characterization of meningococcal carriage and disease isolates in Burkina Faso after mass vaccination with a serogroup a conjugate vaccine

**DOI:** 10.1186/1471-2334-13-363

**Published:** 2013-08-02

**Authors:** Paul A Kristiansen, Absatou Ky Ba, Idrissa Sanou, Abdoul-Salam Ouédraogo, Rasmata Ouédraogo, Lassana Sangaré, Fabien Diomandé, Denis Kandolo, Jennifer Dolan Thomas, Thomas A Clark, Marc LaForce, Dominique A Caugant

**Affiliations:** 1 Norwegian Institute of Public Health, Oslo, Norway; 2 Laboratoire National de Santé Public, Ouagadougou, Burkina Faso; 3 Centre Hospitalier Universitaire Souro Sanou, Bobo-Dioulasso, Burkina Faso; 4 Centre Hospitalier Universitaire Yalgado, Ouagadougou, Burkina Faso; 5 Centre Hospitalier Universitaire Pédiatrique Charles de Gaulle Ouagadougou, Ouagadougou, Burkina Faso; 6 WHO Inter Country Support Team, Ouagadougou, Burkina Faso; 7 Centers for Disease Control and Prevention, Atlanta, USA; 8 Meningitis Vaccine Project, Ferney, France; 9 Faculty of Medicine, University of Oslo, Oslo, Norway

**Keywords:** Neisseria meningitidis, Carriage, Meningitis, Burkina Faso, Conjugate vaccine, MLST, Meningitis belt

## Abstract

**Background:**

The conjugate vaccine against serogroup A *Neisseria meningitidis* (NmA), MenAfriVac, was first introduced in mass vaccination campaigns of the 1-29-year-olds in Burkina Faso in 2010. The aim of this study was to genetically characterize meningococcal isolates circulating in Burkina Faso before and up to 13 months after MenAfriVac mass vaccination.

**Methods:**

A total of 1,659 meningococcal carriage isolates were collected in a repeated cross-sectional carriage study of the 1-29-year-olds in three districts of Burkina Faso in 2010 and 2011, before and up to 13 months after mass vaccination. Forty-two invasive isolates were collected through the national surveillance in Burkina Faso in the same period. All the invasive isolates and 817 carriage isolates were characterized by serogroup, multilocus sequence typing and *porA*-*fetA* sequencing.

**Results:**

Seven serogroup A isolates were identified, six in 2010, before vaccination (4 from carriers and 2 from patients), and one in 2011 from an unvaccinated patient; all were assigned to sequence type (ST)-2859 of the ST-5 clonal complex. No NmA carriage isolate and no ST-2859 isolate with another capsule were identified after vaccination. Serogroup X carriage and disease prevalence increased before vaccine introduction, due to the expansion of ST-181, which comprised 48.5% of all the characterized carriage isolates. The hypervirulent serogroup W ST-11 clone that was responsible for most of meningococcal disease in 2011 and 2012 was not observed in 2010; it appeared during the epidemic season of 2011, when it represented 40.6% of the serogroup W carriage isolates.

**Conclusions:**

Successive clonal waves of ST-181 and ST-11 may explain the changing epidemiology in Burkina Faso after the virtual disappearance of NmA disease and carriage. No ST-2859 strain of any serogroup was found after vaccination, suggesting that capsule switching of ST-2859 did not occur, at least not during the first 13 months after vaccination.

## Background

In the African meningitis belt, a sub-Saharan region stretching from Senegal to Ethiopia [1,2], the populations are affected by annual outbreaks of meningococcal meningitis during the dry season. Devastating epidemics with peak incidence approaching 1,000 cases/100,000 [3] occur every 8–12 years. Most of the epidemics have been attributed to *Neisseria meningitidis* serogroup A (NmA), but some countries have also experienced NmX and NmW epidemics [4,5]. Burkina Faso, a West-African country of approximately 16 million inhabitants, is among the most affected ones, and NmA of sequence types (ST)-5, ST-7 and ST-2859 have been successively responsible for major epidemics [4,6]. In 2002, Burkina Faso was the first African country to experience a serogroup W epidemic, due to the hypervirulent ST-11 clone, probably imported from Saudi Arabia [7-10]. In 2006 a major serogroup X epidemic caused by ST-181 affected the neighbouring country Niger [11,12] and the clone later spread to Burkina Faso [13].

Polysaccharide (Ps) vaccines, used to stop ongoing epidemics [14,15], provide limited and relatively brief protection, do not induce immunological memory and do not protect against carriage [16]. Thus, they have not been successful in preventing the occurrence of new outbreaks. Conjugate vaccines are immunogenic in young children and generate immunological memory. The conjugate vaccines’ ability to prevent carriage acquisition is also another major advantage, as transmission of the bacterium is reduced.

A new NmA polysaccharide-tetanus toxoid conjugated vaccine, MenAfriVac, developed to eliminate NmA epidemics in the meningitis belt, was first introduced on a national scale in Burkina Faso [17-21]. Following a pilot introduction in the district of Kaya in September 2010, the whole 1-29-year-old population in Burkina Faso was vaccinated in December 2010 [21]. The rate of NmA disease and carriage was substantially reduced and herd immunity was generated [22,23]. One of the concerns when introducing a monovalent vaccine is the possibility for serogroup replacement or capsule switch [24-27]. Capsule switch has been documented in several countries [28-32] and the importance of molecular methods in post-vaccination surveillance has been emphasized [29,32,33].

To better understand the impact of implementing a monovalent meningococcal conjugate vaccine in the meningitis belt, including the potential for capsule switching, and to follow the evolution of circulating meningococcal isolates, we characterized invasive and carriage isolates collected in Burkina Faso in 2010 and 2011, before and after MenAfriVac introduction. We present here the molecular characteristics of circulating meningococci up to one year after vaccination.

## Methods

### Ethics

The collection of oropharyngeal samples in Burkina Faso was approved by the Norwegian Regional Committee for Medical Research Ethics, Southern Norway, the Ethical Committee for Health Research in Burkina Faso and the Internal Review Board at Centers for Disease Control and Prevention (CDC), Atlanta, USA. The collection of CSF samples was performed within the Burkina Faso national healthcare system as part of the national surveillance and did not require ethical clearance.

### Meningococcal isolates from healthy carriers

In a repeated cross-sectional study performed in 2010 and 2011 oropharyngeal swabs were collected from a representative proportion of 1-29-year-old residents of three health districts in Burkina Faso; the urban district of Bogodogo, and the rural districts of Dandé and Kaya, during five sampling campaigns, named S5-S9, as described [22,34]. During each of the campaigns, > 5000 individuals aged 1–29 years were enrolled within a 4-week period after their individual written informed consent, or that of their parent or guardian for children below 18 years. A total of 25,521 samples were obtained. Due to an earlier vaccination with MenAfriVac in the district of Kaya (September 2010) than in the rest of the country (December 2010), 3428 of the samples were collected from unvaccinated districts.

Swabbing and laboratory analysis aiming at identifying meningococci were performed by the staff of microbiological laboratories in Burkina Faso: the Centre Hospitalier Universitaire Pédiatrique Charles de Gaulle, Ouagadougou for Bogodogo; the Centre Hospitalier Universitaire Souro Sanou, Bobo-Dioulasso for Dandé; and the Centre Hospitalier Universitaire Yalgado, Ouagadougou for Kaya, as previously described [34]. Isolates suspected to be *N. meningitidis* were sent to Norwegian Institute of Public Health (NIPH) in Oslo, Norway for confirmatory analysis and molecular characterization. All the isolates confirmed as *N. meningitidis* were subject to molecular characterization with the exception of 842 serogroup X isolates collected in the district of Kaya. Due to very high carriage rates of serogroup X meningococci in Kaya about one fourth (273/1115) of the serogroup X isolates were randomly selected for molecular characterization (range, 15.1 - 52.5% per campaign).

### Invasive meningococcal isolates

Cerebrospinal fluid (CSF) samples were collected from 42 cases of meningitis in Burkina Faso, as part of the normal case investigation. CSF samples from the epidemic season of 2010 were sent to the WHO Collaborating Centre for Reference and Research on Meningococci at NIPH in trans-isolate medium [35]. Samples from 2011 were first cultivated at the Centre Hospitalier Universitaire Pédiatrique Charles de Gaulle, Ouagadougou and meningococci were sent to NIPH frozen on dry-ice.

### Characterization of meningococcal isolates

Standard laboratory methods were used to identify meningococci [34] and the serogroup was primarily determined by slide agglutination (Remel, GA, USA). DNA was isolated by suspending 1 loop of bacteria in 200 μl Tris-EDTA (TE) buffer, pH 8.0, heating at 95°C for 10 minutes and centrifugation at 16,000 × g for 5 min. Capsule gene PCR [36] was applied for serogroup determination of isolates non-serogroupable by slide agglutination. Multilocus sequence typing (MLST) using seven housekeeping gene fragments [37] was performed with oligonucleotide primers, as recommended on the MLST website (http://pubmlst.org/neisseria/). Classification by outer membrane protein PorA and FetA variants was done by DNA sequencing of the *porA* and *fetA* genes [38,39]. New MLST alleles, STs, PorA and FetA variants were submitted to the MLST database (http://pubmlst.org/neisseria/). For culture-negative CSF-samples, the genotypic characterization was limited to capsule gene PCR and *porA* sequencing following a nested *porA*-PCR [40].

The invasive isolates were tested for antibiotic susceptibility by determination of minimal inhibitory concentrations (MIC) of penicillin G, ciprofloxacin, ceftriaxone, rifampicin, tetracycline, chloramphenicol and sulphonamides using Etest (AB Biodisk, Solna, Sweden). The isolates were classified as susceptible, intermediate or resistant according to the breakpoints from the European Committee on Antibiotic Susceptibility Testing (http://www.eucast.org).

## Results

### Sample collection

A total of 1,659 meningococcal carriage isolates was retrieved, of which 112 were from unvaccinated districts in 2010 and 1,547 from vaccinated districts in 2010–2011. From a total of 1,205 isolates originating from Kaya, 1,115 were NmX. Of these a subset of 273 isolates was genotyped: 80 from sampling S5, 50 from S6, 33 from S7, 62 from S8 and 48 from S9, representing 20.2%, 17.5%, 15.1%, 52.5% and 51.6% of the NmX isolates, respectively. All the isolates from Bogodogo (158) and Dandé (296) and all the non-NmX isolates from Kaya (90) were subject to genotypic characterization. Hence, a total of 817 carriage isolates was genetically characterized. The number of characterized isolates by campaign and site is shown in Table 1.

**Table 1:** Number of isolates subjected to molecular characterization by district and sampling campaign

**Sampling campaign**	**S5**	**S6**	**S7**	**S8**	**S9**	**Total**
District						
Bogodogo	49 ^a)^	22	38	31	18	158
Dandé	63 ^a)^	72	48	71	42	296
Kaya	103	72	48	80	60	363
Total	215	166	134	182	120	817

^a)^ Unvaccinated district at the time of S5.

Forty-two invasive isolates from Burkina Faso were sent to NIPH, 24 from 2010 before the vaccination campaign and 18 from 2011. Of these, 28 were recovered by culture and could be analyzed by MLST, *porA* and *fetA* sequencing. The remaining 14 samples were tested by capsule gene PCR and *porA* sequencing.

### Molecular characterization of carriage isolates

The 817 carriage isolates comprised 407 NmX (49.8%), 220 NmY (26.9%), 102 NmW (12.5%), 78 (9.5%) nonserogroupable Nm (NmNG), 5 NmC (0.6%), 4 NmA (0.5%) and a single NmB isolate (Table 2). The isolates were assigned to 28 different STs of which 20 belonged to 11 defined ST-complexes (Additional file 1: Table S1). We identified 4 new alleles for the housekeeping genes included in the MLST scheme, 8 new STs, 5 new PorA variants and 4 new FetA variants, all submitted to the MLST database (http://pubmlst.org/neisseria/).

**Table 2:** Number of genotyped carriage isolates from Burkina Faso before and after MenAfriVac vaccination, by serogroup

	**Pre-vaccination**		**Post-vaccination**			
**District**	**B**	**D**	**B**	**D**	**K**	**Total**
Serogroup						
A	0	4	0	0	0	4
B	0	0	0	0	1	1
C	2	0	1	0	2	5
W	5	5	23	58	11	102
X	29	21	42	42	273	407
Y	5	31	16	114	54	220
NG	8	2	27	19	22	78
Total	49	63	109	233	363	817

Isolates were obtained from the districts of Bogodogo (B), Dandé (D) and Kaya (K).

*NG* nonserogroupable.

ST-181 of the ST-181 clonal complex dominated with 396 (48.5%) isolates (Additional file 1: Table S1). The ST-181 isolates were NmX (n= 388) or NmNG (n=8). The dominant PorA/FetA combination of the ST-181 isolates was P1.5-1,10-1; F1-31 (91.0%), independently of the serogroup. In Kaya, 98.1% of the 273 genotyped NmX isolates were assigned to ST-181, 1.5% to ST-5789 and 0.4% (one isolate) to ST-9359. Both ST-5789 and ST-9359 are single locus variants (SLV) of ST-181 at the *adk* and *abcZ* loci, respectively. When extrapolating the proportion of NmX isolates from Kaya with ST-181 to the total number of NmX isolates recovered in each campaign, as many as 1,092 NmX isolates from that district were likely ST-181. With 58 isolates from Bogodogo and 62 from Dandé the total number of NmX ST-181 isolates was likely 1,212, or 73.1% of the carriage isolates in all three districts.

ST-4375 of the ST-23 complex was the second dominating ST representing 15.9% of the genotyped isolates (Additional file 1: Table S1). It included 129 NmY and 1 NmNG. The ST-23 clonal complex was also represented by a single strain assigned to ST-9353, a SLV of ST-4375 at the *adk* locus and first identified in this study. Within the ST-23 complex, the dominating PorA/FetA combination was P1.5-1,2-2; F5-8 (96.9%) independently of ST and serogroup.

Isolates assigned to ST-2881 of the ST-175 complex represented 9.2% of the isolates and were serogrouped as W (n=52), Y (n=12) or NG (n=11) (Additional file 1: Table S1). Among the ST-2881 isolates, 84% expressed the PorA variant P1.5-1,2-36, 92% the FetA variant F5-1 and 76% the combination P1.5-1,2-36; F5-1. The ST-175 complex was also represented by the ST-8638 expressing either a W (n=4) or Y (n=1) capsule. All the ST-8638 isolates were characterized as P1.5-1,2-36; F5-1.

ST-767 of the ST-167 complex included 56 NmY, 1 NmB, 1 NmX and 4 NmNG isolates and accounted for 7.6% of the genotyped strain collection (Additional file 1: Table S1). The PorA/FetA combination P1.5-1,2-2; F5-8 was expressed by 88.7% of the ST-767 isolates. Fifteen of 16 isolates assigned to the two other STs of the ST-167 clonal complex, the ST-2880 and the ST-7375 expressed the same PorA/FetA combination.

A total of 42 (5.1%) ST-11 isolates were found, of which 41 were NmW and one was NmNG (Additional file 1: Table S1). The ST-11 complex was also represented by two ST-9358 isolates identified in the district of Dandé during the sampling campaign S8. ST-9358 is a SLV of ST-11 at the *abcZ* locus. All the ST-11 complex isolates were P1.5,2. Fet A variant F1-1 was found for 95.6% of them and F5-4 on the remaining two isolates. In contrast to other major STs that were found in all three districts, ST-11 was only present in the districts of Bogodogo and Dandé.

NmA carriage was only found in Dandé before vaccine introduction. Four isolates were identified and all were ST-2859; P1.20,9; F3-1. None of the isolates assigned to ST-2859 or classified as P1.20,9 or F3-1 expressed any other serogroup than A.

### ST distribution of carriage isolates over time

**Figure 1:**
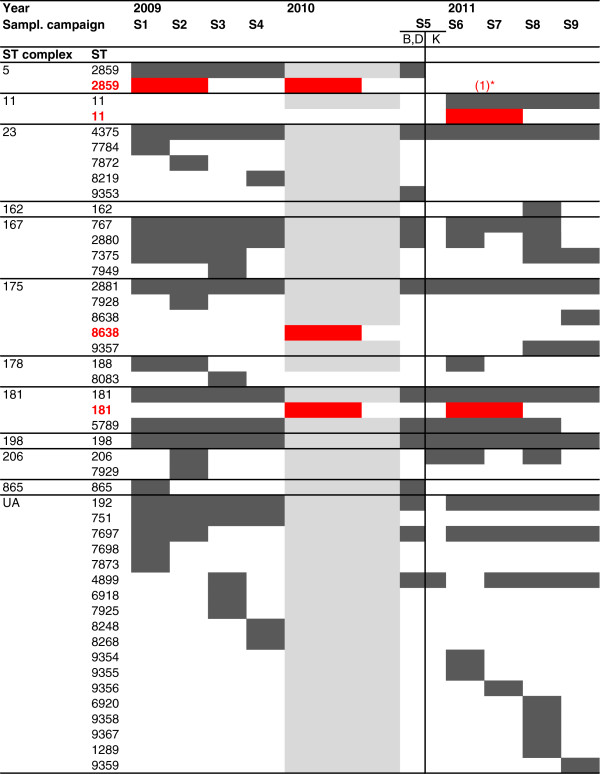
**Genotypes of meningococcal carriage and invasive isolates from Burkina Faso before and after MenAfriVac vaccination.** The figure represents the time points when different sequence types (ST) of carriage isolates (dark grey) and invasive isolates (red) were detected in the carriage study or national surveillance [41], respectively. Each ST was either detected or not detected (blank), or data was unavailable (grey). Horizontal lines separate ST complexes. The vertical line represents vaccination: during sampling campaign S5, the districts of Bogodogo (B) and Dandé (D) were not yet vaccinated while the Kaya district (K) had introduced the vaccine. UA, unassigned to any ST complex. * One single case of serogroup A disease was reported in 2011.

**Figure 2:**
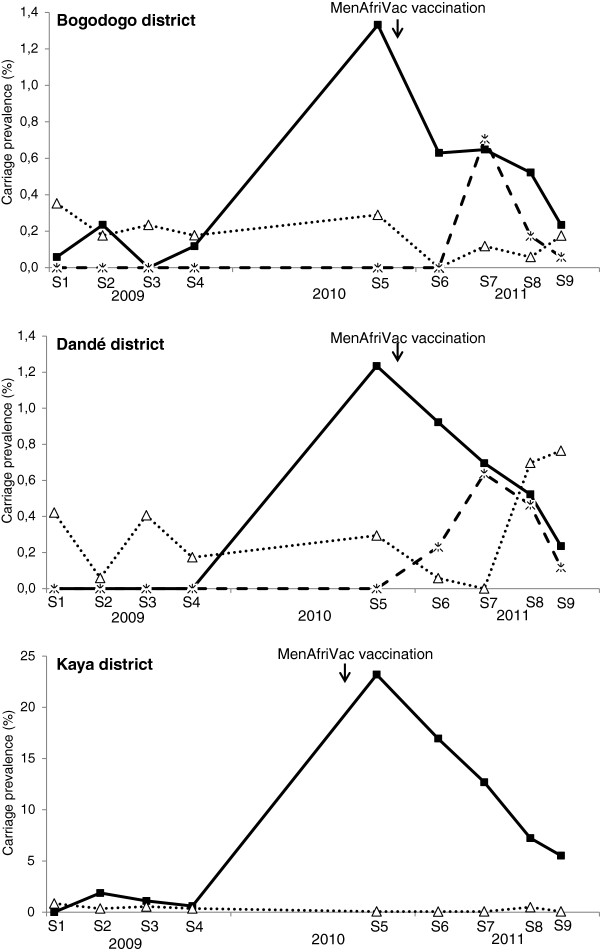
**Carriage prevalence of *Neisseria meningitidis *serogroup X ST-181 and serogroup W ST-11 and ST-2881 in three Districts of Burkina Faso during the sampling campaigns S1-S9, 2009–2011.** Data from S1 to S4 are from a previously published study [34]. Black lines with boxes, serogroup X ST-181. Dotted lines with triangle, serogroup W ST-2881. Dotted lines with cross, serogroup W ST-11.

ST-2859, expressing a serogroup A capsule, was present in the non-vaccinated district of Dandé in October-November 2010, but was not seen after introduction of MenAfriVac (Figure 1). The other two genotypes known to cause outbreaks in sub-Saharan Africa, ST-181 and ST-11, were present in carriers after vaccine introduction. However, NmX ST-181 was circulating already since 2009 [34], while NmW ST-11 was detected only after vaccine introduction (Figure 1). The NmW ST-11 clone was first detected in the district of Dandé from S6 and then in Bogodogo from S7 (Figure 2). A non-groupable ST-11; P1.5-2; F1-1 isolate was also detected in Bogodogo during sampling S6.

Among the NmW isolates 40.6% were ST-11 and 50.5% were ST-2881. The distribution of the two dominant genotypes of NmW isolates was not constant in time. ST-11 carriage peaked at S7 in the districts of Bogodogo and Dandé while carriage of ST-2881 increased from S8 onwards (Figure 2).

### Molecular characterization of invasive isolates

The invasive isolates belonged to 5 STs (Table 3). All the NmA isolates were ST-2859; P1.20,9; F3-1. A single serogroup A isolate from a non-vaccinated 10-year old child was found after vaccine introduction. The NmX isolates were all ST-181; P1.5-1,10-1; F1-31 or F5-69. Fourteen culture-negative samples were P1.5-1,10-1 and likely represented the same NmX clone. Before introduction of MenAfriVac, a single NmW was analysed and characterized as ST-8638; P1.5-1,2-36; F5-1. After vaccine introduction, all the NmW isolates were ST-11; P1.5,2; F1-1.

**Table 3:** **Molecular characteristics of 42 invasive *N. meningitidis *strains recovered from Burkina Faso in 2010-2011**

**Year**	**Culture**	**Serogroup**	**CC**	**ST**	**PorA**	**FetA**	**No. of isolates**
2010	Pos.	A	5	2859	P1.20,9	F3-1	2
	Pos.	W	175	8638	P1.5-1,2-36	F5-1	1
	Pos.	X	181	181	P1.5-1,10-1	F1-31	6
	Pos.	X	181	181	P1.5-1,10-1	F5-69	1
	Neg.	ND	ND	ND	P1.5-1,10-1	ND	14
2011	Pos.	A	5	2859	P1.20,9	F3-1	1
	Pos.	W	11	11	P1.5,2	F1-1	7
	Pos.	X	181	181	P1.5-1,10-1	F1-31	4
	Pos.	X	181	181	P1.5-1,10-1	F5-69	6

*CC* Clonal Complex, *ST* Sequence type, *Pos.* Culture positive, *Neg.* Culture negative, *ND* not determined.

When comparing the genotypes of carriage isolates with those of invasive isolates, we found that the clones responsible for disease were the dominant genotypes of the corresponding serogroups among the carriage isolates; ST-2859; P1.5-1,2-2; F3-1 for NmA, ST-181; P1.5-1,10-1; F1-31 for NmX, and ST-11; P1.5,2; F1-1 for NmW.

### Antibiotic susceptibility of invasive isolates

All the 28 culture positive isolates were susceptible to ciprofloxacin (MIC range, 0.002 - 0.008), ceftriaxone (MIC range, <0.002 - 0.003), rifampicin (MIC range, 0.004 - 0.19), and chloramphenicol (MIC range, 0.5 - 2). Five serogroup W isolates were of intermediate resistance to penicillin G, all the remaining isolates were susceptible. The serogroup A isolates were resistant to tetracycline (MIC range 3 - 4) while the other serogroups were not (MIC range, 0.125 - 0.19). All serogroup A and W isolates were resistant to sulphonamides (MIC 24 - >1024).

## Discussion

In this study we present the molecular epidemiology of meningococcal carriage isolates collected in three districts in Burkina Faso immediately before and up to 13 months after the introduction of a serogroup A conjugate vaccine, in comparison with isolates recovered from patients in 2010 and 2011. Our study showed that the relatively high incidence of NmX disease in 2010–2011 was caused by the same clone (ST-181) found among carriers and patients before vaccination, while the hypervirulent NmW ST-11 clone seemed to have been reintroduced in Burkina Faso after mass vaccination. We did not find evidence that the NmA ST-2859 clone underwent a capsule switch up to 13 months after vaccination either among isolates from carriers or patients.

The collection of invasive strains used to characterize meningococcal disease in Burkina Faso was not a complete or systematic collection taking into account the disease incidence or the geographic distribution of cases. Of 6732 suspected cases of meningitis reported in Burkina Faso in 2010, only 467 CSF samples were analyzed; 130 samples were Nm [41] and of those, 24 isolates sent to the WHO reference laboratory in Oslo. As a result of improved surveillance [23,42], of the 3875 cases reported in 2011, as many as 3125 CSF samples were analyzed; 257 samples were Nm, but only 18 Nm isolates were sent to the WHO reference laboratory. PCR was introduced in Burkina Faso for enhanced laboratory surveillance after vaccine introduction, which may explain the lesser number of collected isolates. Although the strain collection is not completely representative for the meningococcal disease epidemiology in Burkina Faso, it provides a valuable source of information, supplementing other research studies in the same area [13].

The carriage isolates were collected in a multicenter repeated cross-sectional study among 1-29-year-olds in three districts in Burkina Faso. Efforts were made to obtain carriage data from a representative portion of the population, but when comparing these results with the characteristics of invasive isolates, one should keep in mind that the invasive isolates were not necessarily retrieved from the same districts. The carriage study included only people in the same age group as that targeted for vaccination, so this study does not describe characteristics of carriage isolates in < 1 year-olds or > 29-year-olds. A small number of the sampled individuals had not been vaccinated with MenAfriVac and the impact of mass vaccination on NmA transmission also in non-vaccinated has been described previously [22].

The genetic diversity of carriage isolates was low and comparable to that found in 2009 [34], as 96% of the isolates in both studies were assigned to only 11 different STs. Low genetic diversity of meningococcal carriage isolates has been found also in other African countries [43-45], while the diversity is much higher in Europe [46].

In the post-vaccination period up to 13 months after MenAfriVac vaccination, the most striking events were the disappearance of NmA ST-2859, the dominance of NmX ST-181 and the re-emergence of NmW ST-11. Our results show that the NmA isolates circulating in unvaccinated districts in October-November 2010 were identical to those circulating in 2009 [34] and those responsible for NmA disease since 2003 [4,5]. After vaccination, NmA carriage and disease were significantly reduced in 2011 [22,23] and this positive trend continued as no cases of NmA disease were reported in Burkina Faso in 2012 and after the epidemic season of 2013 [41].

In 2009 NmX carriage prevalence in Burkina Faso was 0.44% and the majority of isolates was assigned to ST-181 [13]. NmX carriage was almost exclusively detected in the eastern districts of Bogodogo and Kaya, with a significant increase of prevalence in Kaya during the 2009 epidemic season [13]. The strong increase of NmX ST-181 among carriers and patients in all three districts in 2010 and 2011 and a particularly high carriage prevalence of this clone in Kaya is consistent with a spread from the east to the west after an outbreak in Niger in 2006 [11]. The significant increase of post-vaccination carriage has been shown to be independent of MenAfriVac vaccination as both NmX carriage and disease increased in 2010 before vaccine introduction [22,23]. We here confirm that the increase was due to the ST-181 clone already circulating in 2009 [34].

Our study showed that, after vaccine introduction NmW ST-11 re-emerged among carriers as well as patients. The carriage prevalence of NmW in Burkina Faso in 2009 was 0.34% and all the isolates belonged to the ST-175 clonal complex; the majority assigned to ST-2881 [34]. During the sampling campaign S5 in October-November 2010 NmW was found only in Bogodogo and Dandé and all the isolates were still ST-2881. In 2010, before vaccination, only 8 NmW invasive isolates were identified in Burkina Faso [41] and the only one analyzed by molecular methods was assigned to the ST-175 clonal complex (Table 1). NmW ST-11 which was last seen in Burkina Faso in 2006 [5] reappeared after the country-wide mass vaccination when it was identified in both carriers and patients. Although NmW ST-2881 has been reported to cause meningococcal disease [13,45], until now, the potential to cause large NmW outbreaks has been associated with the hypervirulent ST-11 clone [9].

The circulation of NmW ST-11 exclusively in the districts of Bogodogo and Dandé might be related to the very high carriage prevalence of NmX in Kaya, where up to 23.6% of the individuals were NmX carriers [22]. As NmW ST-11 was first recovered in Dandé in February-March 2011 and then in Bogodogo in May of the same year, another possibility is that the re-introduction of ST-11 happened from Mali, as the Dandé district is close to Mali, and the same NmW ST-11 clone caused disease in Mali in 2007 and 2009 [5,47]. Surveillance data from 2012 showed that NmW disease further increased in Burkina Faso and other countries in the meningitis belt having introduced MenAfriVac [41].

It could be speculated that the change in epidemiology from A to W disease might be attributed to a serogroup replacement caused by the major reduction of NmA carriage and disease incidence. Because pre-vaccination NmA carriage was extremely low [34], vaccine-induced serogroup replacement is unlikely to occur. Epidemic waves have been shown to radically change the meningococcal disease epidemiology in many countries [48]. The last NmW outbreak in Burkina Faso was 10 years ago and the re-emergence of this serogroup fits well with the cyclic reappearance of larger epidemics [8,41].

One concern when introducing a monovalent vaccine is that virulent isolates expressing the capsule targeted by the vaccine may take up genes coding for another capsule by horizontal gene transfer and evade the immune system [30,31,49]. Capsule switched isolates have been shown to conserve their virulence [29]. Up to 13 months after MenAfriVac vaccination, we did not observe any capsule switch among the ST-2859 isolates studied. As the MenAfriVac conjugate vaccine affected transmission of the clone [22] the likelihood that the virulent clone would acquire capsule genes from another meningococcal isolate was probably decreased. As capsule switch might occur any time after vaccine introduction, it is important to continue molecular characterization of isolates retrieved from national surveillance and carriage studies.

## Conclusion

In this study we found that the increase of NmX carriage and disease in Burkina Faso in 2010 and 2011 was due to ST-181 and that the NmW ST-11 clone was probably re-introduced in 2010. Considering the low pre-vaccination carriage prevalence of NmA [34], these observations suggest successive clonal waves of ST-181 and ST-11 contributed to the increase of NmX and NmW disease after MenAfriVac mass vaccination [41], rather than being a result from the significant decrease in NmA disease and carriage [22,23]. The epidemic ST-2859 clone was the only one to cause NmA infections before MenAfriVac vaccination, and no ST-2859 strain of any serogroup was found after vaccination, suggesting that capsule switching of ST-2859 did not occur, at least yet. After a successful implementation of MenAfriVac throughout the whole meningitis belt, the time of devastating NmA epidemics will hopefully be over. However, continued surveillance of disease and carriage and molecular characterization of meningococcal isolates is needed to monitor the long-term impact of vaccination, follow the genetic evolution and antibiotic susceptibility of meningococci, and to improve vaccine strategies. Further development of effective and affordable vaccines against the emerging serogroups X and W should be prioritized. The polysaccharide-conjugated vaccine approach has been successful, but other vaccine strategies should also be explored. The low genetic diversity of meningococcal disease-causing isolates in sub-Saharan Africa and the stability of sub-capsular antigen composition over time, suggest that such antigens could be considered in future vaccine formulations, in addition to conjugate vaccines.

## Competing interest

The authors declare that they have no competing interests.

## Authors’ contribution

PAK, IS, RO, LS, FD, DK, JDT, TAC, MLF and DAC participated in the design of the study. AKB, IS, ASO, SN, RO, LS were responsible for collecting carriage isolates. RO and DK provided the clinical isolates. PAK and FD were responsible for coordination of the carriage study. PAK and JDT contributed with training and supervision. PAK analyzed the data and drafted the manuscript. DAC conceived the study and was responsible for the molecular analysis. All the authors revised the manuscript and approved the final version.

## Pre-publication history

The pre-publication history for this paper can be accessed here:

http://www.biomedcentral.com/1471-2334/13/363/prepub

## Supplementary Material

Additional file 1: Table S1 Molecular characteristics of 817 *N. meningitidis* strains colonizing 1-29-year-olds in Burkina Faso in 2010–2011. Click here for file
