# Supplementary material for: Phenotypic and genotypic characterization of meningococcal carriage and disease isolates in Burkina Faso after mass vaccination with a serogroup a conjugate vaccine
Source: BMC Infect Dis. 2013 Aug 2;13:363. doi: 10.1186/1471-2334-13-363 (PMC3750508; doi:10.1186/1471-2334-13-363)
Supplement: Additional file 1: Table S1 — Molecular characteristics of 817 N. meningitidis strains colonizing 1-29-year-olds in Burkina Faso in 2010–2011. [file 1471-2334-13-363-S1.pdf]

Supplementary file 1

Serogroup	ST-complex	ST no	PorA	FetA	No. of isolates
A	5	2859	P1.20,9	F3-1	4
B	167	767	P1.5-1,10-8	F1-31	1
C	41/44	206	P1.7-43,30	F5-121	2
C	41/44	206	P1.7-2,30-11	F5-2	1
C	865	865	P1.5-1,2-2	F1-6	2
W	11	11	P1.5,2	F1-1	40
W	11	11	P1.5,2	F4-5	1
W	11	9358	P1.5,2	F1-1	2
W	175	2881	P1.5-1,2-36	F5-1	41
W	175	2881	P1.5-1,2-36	F4-28	6
W	175	2881	P1.5-1,2-74	F5-1	4
W	175	2881	P1.5-1,10-1	F5-1	1
W	175	8638	P1.5-1,2-36	F5-1	4
W	175	9357	P1.5-1,2-36	F5-1	2
W	178	188	P1.19,15	F3-9	1
X	162	162	P1.7-2,4	F5-9	1
X	167	767	P1.5-1,10-8	F1-3	1
X	181	181	P1.5-1,10-1	F1-3	1
X	181	181	P1.5-1,10-1	F1-31	346
X	181	181	P1.5-1,10-1	F1-62	2
X	181	181	P1.5-1,10-1	F4-23	3
X	181	181	P1.5-1,10-1	F4-28	1
X	181	181	P1.5-1,10-1	F4-5	1
X	181	181	P1.5-1,10-1	F4-6	1
X	181	181	P1.5-1,10-1	F5-69	26
X	181	181	P1.5-1,10-1	F5-8	1
X	181	181	P1.5-1,10-1	F5-88	1
X	181	181	P1.5-1,10-1	F5-122	1
X	181	181	P1.5-1,10-1	F5-123	1
X	181	181	P1.5,2	F1-31	1
X	181	181	P1.5-1,2-2	F1-31	1
X	181	181	P1.5-3,10-1	F1-31	1
X	181	5789	P1.5-1,10-1	F3-3	1
X	181	5789	P1.5-1,10-1	F4-23	15
X	181	9359	P1.5-1,10-1	F1-31	1
Y	167	767	P1.5-1,10-8	F1-3	51
Y	167	767	P1.5-1,10-8	F1-31	1
Y	167	2880	P1.5-1,10-8	F1-3	4
Y	167	7375	P1.5-1,10-1	F1-3	1
Y	167	7375	P1.5-1,10-8	F1-3	11
Y	175	2881	P1.5-1,2-36	F5-1	10
Y	175	2881	P1.5-1,2-73	F5-1	2
Y	175	8638	P1.5-1,2-36	F5-1	1
Y	23	4375	P1.5,2	F5-8	1

Y	23	4375	P1.5-1,2-2	F5-8	123
Y	23	4375	P1.5-1,2-2	F5-28	1
Y	23	4375	P1.5-1,ND	F5-8	1
Y	23	4375	Neg.	F5-8	3
Y	23	9353	P1.5-1,2-2	F5-8	1
Y	UA	192	P1.18-11,42-1	Neg.	1
Y	UA	7697	P1.18-11,42-1	Neg.	3
Y	UA	9367	P1.21-15,16	F1-7	1
NG	11	11	P1.5-2	F1-1	1
NG	167	767	P1.5-1,10-8	F1-3	2
NG	167	767	P1.5-1,10-8	F1-31	1
NG	167	767	P1.5-1,10-8	Neg.	1
NG	175	2881	P1.5-1,2-36	F5-1	6
NG	175	2881	P1.5-1,2-74	F5-1	5
NG	178	188	P1.19,15	F3-9	1
NG	181	181	P1.5-1,10-1	F1-31	7
NG	181	181	P1.5-1,10-1	Frameshift	1
NG	198	198	P1.18,25	F5-5	5
NG	198	198	P1.18,25-47	F5-5	5
NG	198	198	P1.ND,25	F5-5	1
NG	23	4375	P1.5-1,2-2	F5-8	1
NG	41/44	9354	P1.7-2,9	F1-5	1
NG	UA	192	P1.18-11,42	Neg.	2
NG	UA	193	P1.18-11,42-1	Neg.	11
NG	UA	194	P1.18-11,42-8	Neg.	3
NG	UA	195	P1.18-11,42-9	Neg.	1
NG	UA	1289	P1.22,14-25	F5-5	1
NG	UA	4899	P1.21-14,28-3	F5-66	8
NG	UA	6920	P1.22-11,15-34	F6-5	1
NG	UA	7697	P1.18-11,42-1	Neg.	9
NG	UA	7697	P1.18-11,42-10	Neg.	1
NG	UA	9355	P1.21-14,28-3	F1-3	1
NG	UA	9356	P1.21-14,28-3	F5-66	1

NG: Nonserogroupable
UA: Unassigned
ND: Not Determined
Neg: Negative
Frameshift: Frameshift mutation
